# Gene × environment interactions for ADHD: synergistic effect of 5HTTLPR genotype and youth appraisals of inter-parental conflict

**DOI:** 10.1186/1744-9081-6-23

**Published:** 2010-04-16

**Authors:** Molly Nikolas, Karen Friderici, Irwin Waldman, Katherine Jernigan, Joel T Nigg

**Affiliations:** 1 Department of Psychology, Michigan State University, East Lansing, Michigan, USA; 2 Department of Microbiology and Molecular Genetics, Michigan State University, East Lansing, Michigan, USA; 3 Department of Psychology, Emory University, Atlanta, Georgia, USA; 4 Department of Psychiatry, Oregon Health and Science University, Portland, Oregon, USA

## Abstract

**Background:**

Serotonin genes have been hypothesized to play a role in the etiology of attention-deficit hyperactivity disorder (ADHD); prior work suggests that serotonin may interact with psychosocial stressors in ADHD, perhaps via mechanisms involved in emotional dysregulation. Because the development of behavioral and emotional regulation depends heavily both on the child's experience within the family context and the child's construals of that experience, children's appraisals of inter-parental conflict are a compelling candidate potentiator of the effects of variation within the serotonin transporter gene promoter polymorphism (5HTTLPR) on liability for ADHD.

**Method:**

304 youth from the local community underwent a multi-informant diagnostic assessment procedure to identify ADHD cases and non-ADHD controls. Youth also completed the Children's Perception of Inter-Parental Conflict (CPIC) scale to assess appraisals of self-blame in relation to their parents' marital disputes. The trialleic configuration of 5HTTLPR (long/short polymorphism with A> G substitution) was genotyped and participants were assigned as having high (La/La N = 78), intermediate (La/Lg, La/short, N = 137), or low (Lg/Lg, Lg/short, short/short, N = 89) serotonin transporter activity genotypes. Teacher reported behavior problems were examined as the target outcome to avoid informant overlap for moderator and outcome measures.

**Results:**

Hierarchical linear regression analyses indicated significant 5HTTLPR × self-blame interactions for ADHD symptoms. Examination of the interactions indicated positive relations between reports of self-blame and ADHD symptoms for those with the high and low serotonin activity genotypes. There was no relation between self-blame and ADHD for those with intermediate activity 5HTTLPR genotypes.

**Conclusion:**

Both high and low serotonergic activity may exert risk for ADHD when coupled with psychosocial distress such as children's self-blame in relation to inter-parental conflict. Results are discussed in relation to the role of serotonin in the etiology of the ADHD and related externalizing behaviors.

## Background

Attention-deficit hyperactivity disorder (ADHD) is one of the most commonly diagnosed disorders of childhood and can persist into adulthood. The symptoms of ADHD likely reflect the interplay of complex developmental processes involving both genetic liability and family environmental factors that are believed to shape the gradual, yet staged development of emotional and behavioral regulation during early to middle childhood [1]. Whereas the interplay of these processes is emphasized theoretically, empirical work has mostly examined the main effects of genetic and environmental influences on ADHD separately. Yet, examination of *interactions *between specific genetic and family environmental risk factors is sorely needed in order to test hypotheses regarding their multiplicative versus additive roles in the development of ADHD via behavioral and emotional dysregulation in children [1].

Examination of gene × environment interactions (G × E) is one straightforward way to evaluate these dynamic theories of ADHD. G × E effects may be characterized as genetically-modulated individual differences in sensitivity to environmental risk factors, such that particular environments exert risk for a disorder only for individuals with specific genetic variants or for given genotypes [2]. Quantitative behavioral genetic research has consistently shown that genetic contributions to ADHD are moderate to large [3]. The association of specific candidate genes with ADHD has been replicated [4,5], but effects account for only a small fraction of the heritable component. Behavioral geneticists have long known that that the genetic and non-shared environmental variance components in traditional behavioral genetic models contain main effects as well as G × E interactions [6]. Therefore, the very high heritability seen in ADHD might be evidence in favor of G × E interactions for the disorder. Additionally, several environmental risk factors for ADHD have been identified [7,8]. In all, the notion of G × E effects operating for ADHD is a compelling empirical possibility [9] in addition to its aforementioned theoretical appeal.

The next steps involve selection of specific genetic markers and specific family environment risk factors that may be operating synergistically in the development of behavioral and emotional regulatory capacities, deficiencies of which are believed to contribute to ADHD symptoms. Because of the hazards of arbitrary selection of variables, selection of the genes and environmental variables to study require theoretical as well as empirical considerations. We consider candidate genes first, then candidate environments.

### Selection of candidate gene for G × E in ADHD

Genes of the dopaminergic and noradrenergic neurotransmitter systems have been most studied due to their presumed centrality in the action of psychostimulants and noradrenergic reuptake inhibitors [10]. However, they have been primarily associated theoretically with ADHD's cognitive and reward-processing elements, rather than emotional dysregulation [11]. Genes coding for proteins involved in emotional regulation and impulse control systems are needed to test an integrated regulation model of ADHD [1,11].

A particularly attractive neural system from this perspective is the serotonergic system. It has been hypothesized to play a role in ADHD because of its association with externalizing problems such as impulse control and aggression [12,13]. Furthermore, serotonin is central to classical theories of behavioral regulation and constraint [14]. At the genetic level, the serotonin transporter gene is expressed in brain regions often implicated in emotion regulation, attention, and motor control [15].

We hypothesized that the serotonin transporter gene plays a role in the development of emotional and behavioral regulation and so is relevant to G × E effects in ADHD. The functional 44-bp promoter polymorphism of the serotonin transporter gene (5HTTLPR) has been frequently studied for psychiatric conditions. The "short" allelic variant results in reduced transcription efficiency and lower uptake activity [16,17]. In contrast, the "long" (or "high activity" allele) has been associated with ADHD in case-control and within-family studies [4]. However a recent meta-analysis indicated significant heterogeneity in effect sizes across studies [5].

One potential explanation for that heterogeneity is that unmeasured environments are serving to enhance (or attenuate) the genetic association with the disorder. G × E investigations have been encouraged by evidence that the genetic regulation of serotonin neurotransmission is sensitive to experiences [18,19]. In addition, a recent genome-wide investigation of G × E interactions for ADHD found suggestive (although not genome-wide significant) evidence of an interaction between parental warmth and a SNP in the serotonin transporter gene (p = 0.008) [20]. Thus, serotonin genes and 5HTTLPR specifically may be particularly well-suited for investigation of G × E in relation to a regulatory model of ADHD.

### The role of serotonin in ADHD

Despite the appeal of 5HTTLPR as a model micro-system for examining inputs to regulatory problems and ADHD, the role of serotonin requires further comment. First, as noted above, the 5HTTLPR "long" allele has been associated with ADHD, but the "short" allele with conditions that are often comorbid with ADHD, including mood problems, disruptive behavior problems, persistent aggression, and conduct disorder [21-24]. Likewise, studies of peripheral and central serotonergic functioning in children also have produced somewhat contradictory results. Both low [25-28] and high [29-31] serotonergic functioning have shown relationships with impulsivity and aggression in children and adolescents, whereas studies of animals and adult humans have mainly implicated low serotonergic activity for impulsive and aggressive behaviors [32-35]. These seeming contradictions would benefit from explanation and leave it unclear whether linear effects should be expected.

Second, with regard to nuances of the gene itself, we now know that an A>G substitution is contained in a subset of the repeat sequences of 5HTTLPR. The A>G substitution has functional significance, such that the long allele with the G substitution (Lg) functions similarly to the short allele (i.e., reduced transcription efficiency and decreased expression) [36-38]. This resultant triallelic model of 5HTTLPR (La, Lg, short) failed to show association with ADHD in one study [39], yet the low functioning alleles (short and Lg) were associated with conduct problems using case-control and within-family methods [22].

At least two basic possibilities can be suggested for these discrepant results. 5HTTLPR may function differently for ADHD and cognitive impulsivity versus aggressive impulsivity [40] or, alternatively, for the ADHD symptom dimensions of inattention and hyperactivity [41]. Thus, examination of ADHD as a unidimensional construct, its two constituent symptom domains of inattention and hyperactivity-impulsivity, and comorbid symptoms of oppositional and aggressive behavior, may aid in the clarification of these relationships.

Another, more provocative, possibility is that both the high and low activity 5HTTLPR genotypes confer risk for ADHD (and perhaps for related disruptive behaviors). Supporting this notion, the one prior study examining G × E interactions involving 5HTTLPR and ADHD found an association main effect with the more efficient "long" allele of 5HTTLPR (high serotonin transporter activity), yet an interaction with psychosocial adversity with the low-efficiency "short" allele of 5HTTLPR [42]. That is, it may be that optimal adjustment occurs at the mid-range of serotonergic transcription, and that at either extreme, the child is vulnerable to regulatory dysfunctioning in adverse contexts. This possibility, not formally examined in prior studies, requires that nonlinear as well as linear effects be considered in the triallelic model.

### Selection of environmental risk factors for G × E

The only genome-wide association G × E study of ADHD suggested the serotonin transporter may interact with family distress measures [20]. This is not surprising given that the development of emotional and behavioral regulation in children is dependent on a range of variables related to the family environment. Familial conflict in particular has emerged as a particularly good candidate domain, due to its high salience for children and findings that chronic emotional stress during development can alter cortical functioning [43]. Family distress or conflict has been assessed in numerous ways over many decades of work. However, recent work consistently has emphasized the role of interparental conflict in child adjustment, including not only internalizing but also ADHD and externalizing behavior problems [44-53].

Nonetheless, developmental and family research has indicated that the child's appraisals of interparental conflict (and not just exposure) often play a determining role regarding the impact of conflict on youth behavior problems [44]. That is, the frequency of the conflict may not be as important for the development and persistence of attention and behavior problems as the extent to which children blame themselves or feel threatened by their parents' disagreements. This perspective is particularly sensible in light of our interest in regulatory functions in ADHD [1] which are likely mediated at least in part by emotional arousal and construal. In support of this view, youth reports of interparental conflict are more predictive of behavior problems than are parents' report of their own conflict and marital satisfaction [53,54]. Children's observed distress level while witnessing conflict has been especially associated with biological changes, including increased stress response [55] and alterations in parasympathetic nervous system activity [56]. Additionally, a recent meta-analysis found that cognitive appraisals of self-blame in regard to interparental discord emerged as a particularly salient predictor of children's internalizing and externalizing problems [57]. Thus, construals of self-blame in relation to marital conflict emerges from the literature as a particularly potent experiential moderator that may interact with genetic liability in child dysregulation, and thus guided our hypothesis about how G × E may influence ADHD in the context of a dysregulation conception.

### Summary

The aim of the current study was to examine G × E effects for ADHD within the framework of a dysregulatory conception of ADHD, and thus focused specifically on examining a theoretically relevant interaction between the triallelic 5HTTLPR polymorphisms and youth perception of self-blame in relation to marital conflict. Given the mixed literature as to whether high or low serotonin activity would be a risk factor, the hypotheses included both linear models (that either low *or *high 5HTTLPR activity genotypes confer sensitivity to the psychosocial risk element) and a non-linear model (both low *and *high 5HTTLPR activity genotypes confer sensitivity to environmental risk, whereas moderate levels confer protection).

## Methods

### Participants

Participants were 304 children and adolescents ages 6-18 years (*M *= 14.04, *SD *= 2.70, 56.6% male). The sample was recruited using mass mailings to parents in the local school districts, public advertisements, and outreach to local clinics in order to screen as broad of a range of volunteers as possible for the study (and avoid the inferential biases inherent in a purely clinic referred sample). A multi-stage screening process was used to identify cases and non-cases meeting research criteria among those who volunteered. At stage 1, rule-outs were evaluated by a telephone screen (physical handicap, non-native English speaking, mental retardation, autistic disorder, and prescription of long-acting psychoactive medications--due to affiliated studies of neuropsychological status that required medication washout; stimulant use was not a rule out). Families passed through the telephone screen were then invited to complete the stage 2 diagnostic screen. Informed consent was obtained from all participating parents; children provided written assent. These studies were approved by the local Institutional Review Board.

For stage 2, parents and teachers completed normative behavioral rating scales, including (1) the Conners' (1997) Rating Scale-Revised short form [58], and (2) the DSM-IV ADHD Rating Scale [59]. One parent (in most cases, the mother) completed the Kiddie Schedule for Affective Disorders and Schizophrenia-E (KSADS-E) [60] with a trained master's level clinical interviewer.

At stage 3, final eligibility and diagnostic assignment were made using a best-estimate procedure as follows. Data from the KSADS-E and the parent and teacher rating scales, along with interviewer notes and observations and history of treatment, was presented to a diagnostic team consisting of a board-certified child psychiatrist and a licensed child clinical psychologist. Both professionals arrived independently at a clinical decision regarding ADHD subtype and comorbid diagnoses. Agreement rates were acceptable for all diagnoses (all kappas >.89). In all cases of disagreement, consensus was able to be reached upon discussion.

Those youths with subthreshold ADHD (5 symptoms, N = 10) or situational ADHD (n = 6) were included for analysis of dimensional symptom scores but not in analysis of diagnostic group effects. The final sample consisted of 137 non-ADHD participants, 151 ADHD participants (72 Primarily Inattentive Subtype, 1 Hyperactive-Impulsive Subtype, 78 Combined Subtype), and 16 subthreshold/situational (ADHD NOS).

#### Exclusionary criteria

Youth were excluded if they had mental retardation (based on having a full-scale IQ <75), head injury with a loss of consciousness, a history of seizures as ascertained by parent report, autistic or pervasive developmental disorder as reported by the parent, or KSADS-E diagnosis of current major depressive episode (viewed as rendering ADHD symptom ratings difficult to evaluate), lifetime bipolar disorder, or lifetime psychosis.

### Perception of inter-parental conflict

To assess inter-parental conflict, children and adolescents completed the Children's Perception of Inter-parental Conflict scale (CPIC) [53] with the assistance of a staff person. Youth rated the 48 CPIC items on a three-point scale (0-2: false, sort of true, and true). Children completed the CPIC if they either (1) lived with both biological parents (n = 197), (2) lived with one biological parent/guardian and a step-parent or other co-habitating adult (n = 59), or (3) lived with one biological parent but had frequent contact with their other parent and often observed interactions between their parents to complete the CPIC (co-parenting; n = 48). Children who had never lived with or interacted with a second parent, or no longer had meaningful contact with a second parental figure, were not included. Although the CPIC was developed for children 8 and older [53], one concern was that young children even older than age 8 might have difficulty reading or comprehending the items [61]. Therefore, for children who were under the age of 10 or who were identified as poor readers on the WIAT [62] word reading test earlier in the visit, the examiner read the items aloud while the child looked on. Prior work [63] suggested that this procedure yielded an equivalently valid factor structure for the CPIC in 6-9 year old children versus in children 10 and older.

Factor analytic work [63] concluded that the CPIC item set yields 4 factors. The nine-item self-blame scale was the focus of analysis here based on prior work demonstrating specific links between self-blame and youth behavior problems [48]. Sample items from the CPIC self-blame scale include "My parents usually argue about something that I do"; "It is usually my fault when my parents argue"; and "I am to blame when my parents argue." Internal consistency reliability for the nine-item self-blame scale was satisfactory (alpha = .83). Self-blame scores ranged from 0-18, with 0 indicating denial of all self-blame items (n = 74 youth, 24.3% of the sample, scored a 0). To capture the entire range of self-blame, all 304 youth were retained in the analyses.

Bivariate correlations revealed significant relationships between self-blame and (a) child's report of conflict frequency and severity (r = .34, p < .001) and (b) parent report of the frequency of negative interactions between themselves and their spouses in front of the child as rated on the O'Leary-Porter scale (r = .27, p < .001) [64]. These data suggested that the self-blame scale was indeed tapping into a cognitive and/or emotional appraisal of interparental conflict.

Although self-blame was the primary marital conflict appraisal examined in the current study, to provide a contrasting test we also examined scores on the eleven-item conflict properties scale, which measures the frequency and intensity of observed marital conflict. Items from the conflict properties scale included "I never see my parents arguing or disagreeing" (reverse scored), and "When my parents have an argument, they yell a lot." Internal consistency reliability for the 11-item scale was adequate (alpha = .87). Conflict properties scale scores ranged from 0-22, with a score of zero indicating denial of all conflict properties items (n = 1). Again, to capture the entire range of conflict properties, all 304 youth were included. It was *not *expected that this measure would interact with serotonin genotype. Rather, it provided a contrast measure to evaluate the likelihood that we are detecting interactions on all measures regardless of content, versus detecting interactions specifically for self-blame.

### DNA collection and serotonin transporter genotyping

#### Overview

Buccal DNA samples were requested from all participating children and adolescents and purified using previously used methods [65].

#### Serotonin transporter promoter polymorphism

The 44-bp promoter polymorphism of the serotonin transporter gene (5HTTLPR) and the rs25531 A>G polymorphism were genotyped as follows. The "short and long" alleles of the 5HTTLPR were genotyped according to previous methodology [16] with the following modifications to the primer sets (5'-GACTGAGCTGGACAACCACG-3' and 5'-GGTTGCCGCTCTGAATGCCA-3'). Genomic DNA (40 to 60 ng) was amplified using the *Taq *DNA Polymerase kit (Qiagen Inc., Valencia, CA), standard kit protocol, including 1.5 mM MgCl_2_, 0.2 mM dNTPs, and 0.7 μM primer. Polymerase chain reaction (PCR) conditions consisted of an initial denaturing step at 95°C for 3 minutes, followed by 35 cycles of: 95°C denaturation for 30 seconds, 63°C annealing for 30 seconds, and an extension at 72°C for 45 seconds, followed by a final extension step of 4 minutes at 72°C. A portion of the amplified DNA was analyzed using a 2% agarose gel to determine the L/S alleles. The remainder of the amplification reaction was digested with *Msp *I endonuclease (New England Biolabs, Ipswich, MA) and examined by 3% agarose gel electrophoresis. The final products were (340, 120, and 64 bp) for (La), (174, 166, 120, and 64 bp) for (Lg), and 484 bp (short).

Based on previous work [22,37] we assigned the following genotypes to the high, intermediate and low activity groups. Those homozygous for the La allele were classified as "high" 5HTTLPR activity (n = 78). Those with the La/Lg or La/short genotypes were classified as "intermediate" 5HTTLPR activity (n = 137). Individuals with Lg/Lg, Lg/short, or short/short genotypes were classified as "low" 5HTTLPR activity (n = 89).

### ADHD symptom outcome measures

In order to examine effects at all levels of symptoms and to avoid artifacts associated with examining G × E using dichotomized outcomes [66], we elected to use dimensional ratings of on the Conners' Rating Scale [58] as the primary outcome measures. These included outcomes for the main ADHD analyses (ADHD Index, Cognitive Problems, and Hyperactivity subscales) as well as supplemental analyses for the Oppositionality scale as a contrast measure. We examined raw scores on the Conners' Rating Scale scores as the primary measure, rather than the ADHD Rating Scale, because the distribution of scores better approached normality and thus maximized statistical power. The Conners Rating Scale also allowed us to include measures of ADHD and oppositional behaviors from the same measure.

The primary dependent measures relied on *teacher *ratings for several reasons. First, it enabled complete disaggregation of the sources of data (which could be partially confounded by parent ratings, because parents who are distressed by marital conflict may also inflate their ratings of child ADHD symptoms). Put another way, teacher ratings of child ADHD and oppositional behaviors are unlikely to be directly influenced by inter-parental conflict in the home. Teacher reports of attention problems in particular have also been cited as a robust predictor of life outcomes when controlling for other environmental variables, including single parent status, socially disadvantaged community, parental education, and child IQ as well as disruptive and emotional behavior problems [67-69]. However, *parent *ratings are also reported in order to evaluate strength of internal replication via a second informant.

For both teacher and parent report, interactions were first examined for the total ADHD Index score on the Conners' Rating Scale (alpha = .89). In a Fisherian strategy [70], if that effect was significant then we planned to examine the symptom dimensions of inattention and hyperactivity-impulsivity separately (with p value of .025 as threshold for significance for each dimension in order to adjust for multiple comparisons).

### Statistical analyses

#### Testing gene -environment interplay for ADHD symptoms

Prior to examining G × E effects, we examined gene-environment correlations (rGE). A relation between the genetic and environmental risk factors (e.g., 5HTTLPR and self-blame) can confound any test of G × E, as rGE effects can potentially emerge (falsely) as G × E. We tested for rGE by examining differences in reports of self-blame by level of 5HTTLPR genotype.

Next, hierarchical linear regression analyses were used to examine potential gene × environment interactions for ADHD. As mentioned earlier, we set out to examine both the linear effects of 5HTTLPR (i.e. that either low *or *high serotonin activity genotypes exert risk) and non-linear effects of 5HTTLPR (i.e. that both low *and *high serotonin activity genotypes exert risk). The interactions (linear × self-blame and non-linear × self-blame) were evaluated using the following orthogonal coding system. For the linear effects, high, intermediate, and low activity 5HTTLPR genotypes were coded as 1, 0, -1 respectively. For non-linear effects, high, intermediate, and low activity 5HTTLPR genotypes were coded -1, 2, -1, respectively.

#### Secondary tests of G × E interplay for oppositional and conduct problems

Although our main focus was on ADHD symptoms as the outcome, dysregulation models involving serotonergic functioning and conflict within the family environment have also been advanced for problems commonly comorbid with ADHD, including oppositional and conduct problems. Therefore, as a secondary test of G × E effects, we also conducted the interaction analyses using reports of oppositional problems as the dependent measure in order to determine if effects generalized to disruptive behaviors or were specific to ADHD.

## Results

### Sample characteristics and covariates

Demographic and descriptive statistics of the children (excluding the n = 16 youth with ADHD-NOS) are presented in Table 1. As expected, youth in the ADHD group were rated as having more inattentive and hyperactive symptoms across informants and measurement method. The ADHD group was more predominately male and significantly younger than the non-ADHD group; thus age and gender were covaried in all models. Families of ADHD children also had significantly lower annual incomes than the non-ADHD families.

**Table 1:** Demographics and descriptive statistics for ADHD cases and non-ADHD controls.

	Control (N = 137)	ADHD (N = 151)	p
% Male	48.2	64.2	.006
% Caucasian	74.5	78.1	.46
% African-American	14.6	11.3	.40
% Latino	6.6	3.3	.20
% Other	4.4	7.3	.30
Age (SD)	14.7 (2.4)	13.5 (2.8)	<.001
% Both Biological Parents	71.5	60.3	.044
% Parents Separated/Divorced	10.9	18.5	.071
% Parents Re-Partnered	17.5	21.2	.43
Yearly Household Income (SD)^+^	74 (36)	63 (36)	.02
*KSAD Diagnostics*			
Inattentive Symptoms (SD)	1.2 (2.0)	7.3 (1.8)	<.001
Hyperactive Symptoms (SD)	.57 (1.1)	4.1 (3.2)	<.001
% ODD	8.8	27.2	<.001
% CD	1.5	9.9	.002
% MDD	10.2	21.9	.008
*Conners' Teacher Report*			
Cognitive Problems T Score	51.8 (12.3)	61.7 (14.6)	<.001
Hyperactivity T score	49.8 (9.3)	60.3 (11.8)	<.001
*ADHD Rating Scale -- Teacher Report*			
Inattentive Symptoms	.62 (1.7)	4.0 (3.2)	<.001
Hyperactive Symptoms	.32 (.10)	2.1 (2.8)	<.001
*5HTTLPR Genotype*			
% High Activity (La/La)	.28	.25	.53
% Moderate Activity (La/Lg, La/s)	.42	.46	.49
% Low Activity (Lg/Lg, Lg/s, or s/s)	.30	.29	.88
CPIC Self-Blame Score	1.9 (3.0)	4.1 (3.6)	<.001
CPIC Conflict Properties Score	9.1 (5.5)	11.6 (5.0)	<.001

*Note*. SD = standard deviation; ODD = Oppositional Defiant Disorder; CD = Conduct Disorder; MDD = Major Depressive Disorder-Lifetime. KSADS-E Diagnostics based upon parent report.

+ Income in thousands of dollars.

Children in the ADHD group were less likely to be living with both biological parents than non-ADHD children. This latter difference in regard to family constellation may potentially influence reports of self-blame, as children experiencing parental separation and/or divorce may be more likely to observe frequent and intense marital conflict and therefore, may have higher levels of self-blame. Whereas children who had experienced parental separation and/or divorce reported higher scores on conflict properties (M = 11.8, *SD *= 5.5) and higher mean levels of self-blame (*M *= 3.4, *SD *= 3.5) than those living with both biological parents (conflict properties *M= *10.0, *SD *= 5.2; self-blame *M *= 2.9, *SD *= 3.3), the difference was marginally significant for conflict properties [F(1,302) = 2.7, p = .07) and was not significant for self-blame,[F(1,302) = 1.6, p = .20]. Children living with a step-parent did not report significantly higher levels of conflict properties or self-blame than did children living with two biological parents (ps > .33). Despite this, as a precaution we covaried family composition (i.e., living with two biological parents, living with one biological parent and one step-parent, living with one biological parent only) in the main G × E analyses (with no effect on the results).

Youth in the ADHD group also had significantly higher scores on the conflict properties subscale of the CPIC compared to their non-ADHD counterparts (see Table 1). Although this did not appear to significantly differ based upon family composition, we also conducted the G × E models with conflict properties entered as a covariate (with no significant change in results).

### Ethnicity/race and 5HTTLPR

There were no differences between the ADHD and non-ADHD group in terms of ethnicity or race (see Table 1). However, ethnic variation is a potentially important variable in genetic studies. Table 2 shows that, as in other reports [50,51], there were significant differences in the distribution of 5HTTLPR alleles by ethnic group, such that the La and Lg alleles both occurred more frequently in African-American children than non-African-American children. As a precaution, race and ethnicity were covaried as follows. Ethnicity/race was first divided into three codes: (1) Caucasian versus non-Caucasian, (2) African-American versus non-African-American, and (3) Latino versus non-Latino. The three codes were then entered at step 1 of all regression models.

**Table 2:** 5HTTLPR allele frequencies and genotypes by self-reported ethnic group.

	La	Lg	Short
Caucasian (N = 229)	.49	.06	.45
African-American (N = 42)*	.61	.19	.20
Latino (N = 15)	.33	.07	.60
Other (n = 18)	.28	.17	.55
*Total*	*.48*	*.09*	*.43*

*Note*. *La and Lg allele frequency significantly greater in African-American participants compared to non-African American participants, (La, p = .048; Lg, p = .007).

### Main effects of 5HTTLPR on ADHD

Table 1 also includes the 5HTTLPR allele frequencies in the ADHD and non-ADHD groups. There were no significant differences in the distribution of 5HTTLPR alleles among ADHD and control youth (all *p *> .25). There were also no significant main effects of 5HTTLPR genotype when dimensional measures of ADHD were examined. As seen in Table 3, 5HTTLPR genotype was unrelated to ADHD and externalizing symptoms (KSADS-E) as well as scores on the ADHD Rating Scale and Conners' Rating Scale.

**Table 3:** ADHD and externalizing symptoms: main effect tests of high, intermediate and low activity 5HTTLPR genotypes.

	High	Intermediate	Low	p	p^e^	p^eas^
N	78	137	89			
*KSAD Diagnostics*						
Inattentive Symptoms	5.5 (3.5)	6.0 (3.2)	5.5 (3.7)	.49	.51	.22
Hyperactive Symptoms	3.7 (3.3)	3.8 (3.3)	4.2 (3.3)	.60	.47	.52
ODD Symptoms	1.7 (2.3)	1.6 (2.2)	1.8 (2.4)	.81	.80	.83
CD Symptoms	.33 (.65)	.57 (1.3)	.30 (.87)	.10	.09	.09
*Conners' Teacher Report*						
Cognitive Problems	3.0 (3.5)	3.4 (4.0)	3.8 (4.7)	.47	.45	.44
Hyperactivity	4.3 (4.8)	3.6 (3.8)	3.1 (3.8)	.20	.24	.13
*ADHD Rating Scale Teacher Report*						
Inattention	4.6 (6.7)	4.3 (6.1)	4.9 (6.5)	.95	.96	.93
Hyperactivity	3.1 (5.9)	2.4 (4.5)	2.3 (4.6)	.49	.65	.46

Note. High activity genotypes (La/La); Intermediate activity genotypes (La/Lg, La/short); Low activity genotypes (Lg/Lg, Lg/short, short/short.) p^e ^= ethnicity corrected p value, p^eas ^= ethnicity age, and sex corrected p value. Inattentive, Hyperactive, ODD, and CD symptoms are parent report on the KSADS-E. Conners' Scores and ADHD Rating Scale scores are raw total scores on each measure (higher scores signify symptoms/problems).

### Test for gene-environment correlation

There were no significant differences in reports of self-blame across the three genotype groups (p = .22), suggesting an absence of gene-environment correlation between 5HTTLPR genotype and children's report of self-blame. Lack of correlation between this genetic marker and this environmental measure signals that this particular rGE effect is unlikely to emerge as spurious interaction findings in the present analyses (although correlations among unmeasured genes or environments cannot be ruled out).

### Main analyses of G × E interaction effects

#### Teacher report

##### Conners ADHD index

**Figure 1:**
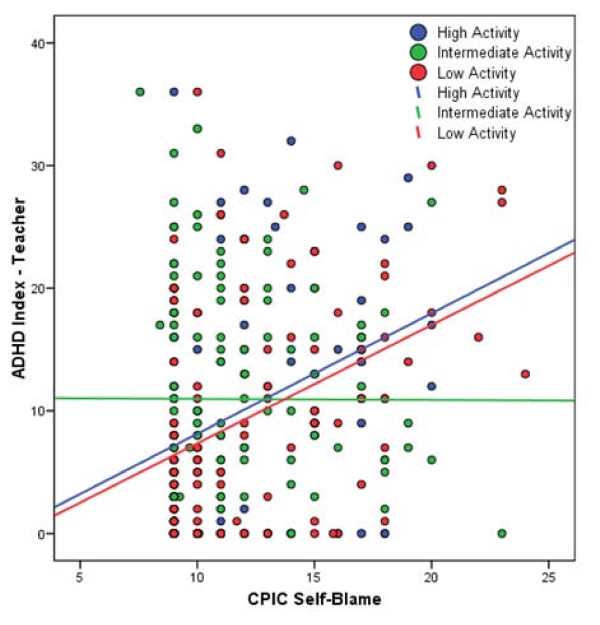
**5HTTLPR × self-blame interaction predicting teacher ADHD index score**. Scatter plot of the CPIC self-blame and ADHD Index (teacher report) data are displayed and color-coded by genotype group. Solid lines represent best-fitting regression line for each genotype group.

Hierarchical linear regression analyses were used to test for G × E effects. All regression models included the following covariates: age, gender, ethnicity, family composition, and overall conflict frequency/intensity (as reported by the child on the CPIC). All modeling results reported herein include these covariates. Results revealed a significant main effect of self-blame for the total ADHD Index raw score [b = .19, 95% confidence interval .08-.29, p = .003, total R^2 ^= .11], indicating an increase in total ADHD symptoms with higher reports of self-blame. The linear (low activity as risk) and non-linear (low and high activity as risk) main effects of 5HTTLPR genotype were nonsignificant as was the linear × self-blame interaction (all ps > .28) However, the non-linear × self-blame interaction was significant [b = .17, 95% confidence interval .06-.24, p < .001, ΔR^2 ^= .02, total R^2 ^= .13]. Examination of the simple slopes revealed that there was no relationship between ADHD symptoms and self-blame for those with the intermediate activity genotypes (r = -.05, p = .53). In contrast, a significant and positive relationship between self-blame and ADHD emerged for those with the high (La/La, r = .32, p < .001) and low (Lg/Lg, Lg/short, short/short, r = .40, p < .001) activity genotypes (See Figure 1).

##### Cognitive problems and hyperactivity

**Figure 2:**
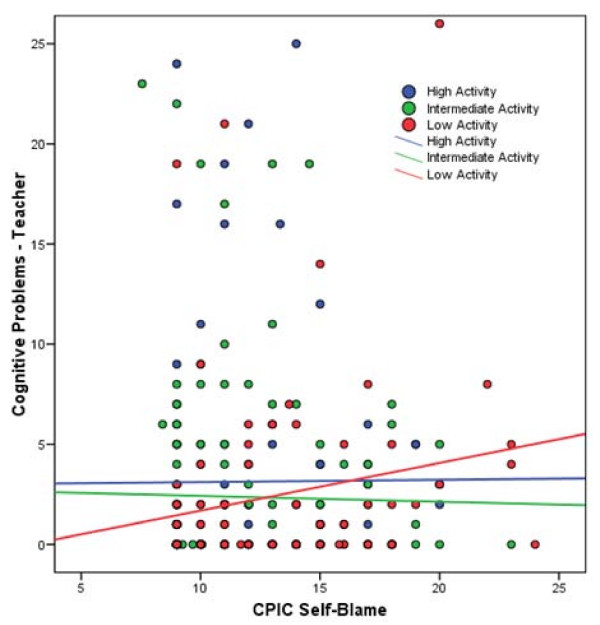
**5HTTLPR × self-blame interaction predicting teacher cognitive problems score**. Scatter plot of the CPIC self-blame and Cognitive Problems (teacher report) data are displayed and color-coded by genotype group. Solid lines represent best-fitting regression line for each genotype group.

We then proceeded to examine the Conners ADHD symptom dimensions of inattention and hyperactivity (measured via the Cognitive Problems and Hyperactivity raw subscale scores on the Conners'). For Cognitive Problems, there was a significant main effect of self-blame [b = .21, 95% confidence interval .09-.32, p < .001, total R^2 ^= .07]. There was no main effect of 5HTTLPR genotype group using either the linear (p = .29) or non-linear (p = .54) coding schemes. The linear × self-blame interaction was nonsignificant (*p *= .54). The non-linear × self-blame interaction showed a trend, but was also not significant [b = .07, 95% confidence interval -.01-.14, p = .08, ΔR^2 ^= .008, total R^2 ^= .08] (see Figure 2).

**Figure 3:**
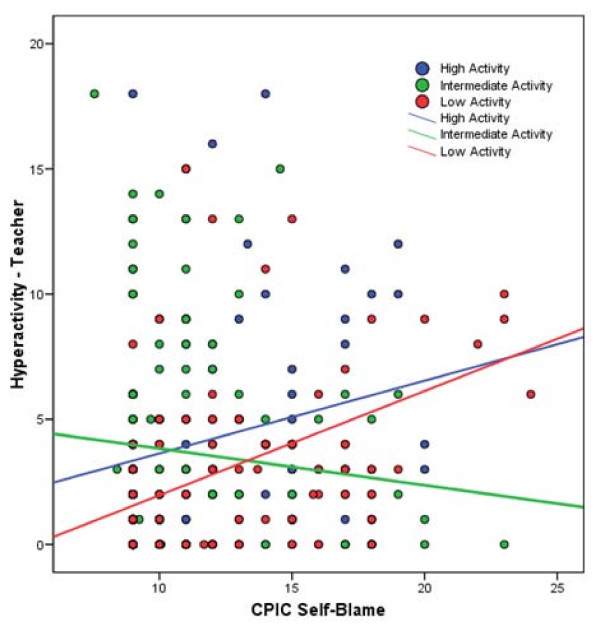
**5HTTLPR × self-blame interaction predicting teacher hyperactivity score**. Scatter plot of the CPIC self-blame and Hyperactivity (teacher report) data are displayed and color-coded by genotype group. Solid lines represent best-fitting regression line for each genotype group.

For Hyperactivity, results mirrored those for the ADHD index. The main effect of self-blame was significant [b = .15, 95% confidence interval .05-.26, p = .005, total R^2 ^= .10]. The linear and non-linear main effects of 5HTTLPR genotype were again nonsignificant as was the linear 5HTTLPR × self-blame interaction (all p > .26). In contrast, the non-linear 5HTTLPR × self-blame interaction was significant [b = .15, 95% confidence interval .08-.23, p < .001, ΔR^2 ^= .02, total R^2 ^= .12]. Examination of the simple slopes revealed a similar pattern of results. For youth with the low and high activity serotonin genotypes, there was a significant and positive relationship between self-blame and Hyperactivity (high 5HTTLPR activity, r = .25, p < .01; low 5HTTLPR activity r = .43, p < .001). Yet, there was no relationship between self-blame and ADHD for those with the intermediate serotonin activity genotypes (r = -.15, p = .16) (see Figure 3).

Overall, for teacher report of ADHD symptoms, interactions indicated significant non-linear G × E effects predicting scores on both the ADHD Index and on the Hyperactivity scales, both of which remain significant after correction for multiple tests (ps < .025). Further, non-linear 5HTTLPR × self-blame interactions remained significant after adjusting for a number of covariates, including gender, age, ethnicity, family composition, and overall level of conflict frequency/intensity (measured via the CPIC conflict properties scale).

#### Parent report

##### ADHD index

To evaluate degree of internal replication of results, G × E regression models were repeated with parent report on the Conners' Rating Scale ADHD index and the two symptom dimension scales with uncorrected p values. For the ADHD index, results again indicated a significant main effect of self-blame [b = .29, 95% confidence interval .19-.40, p < .001, total R^2 ^= .08]. The main effect of 5HTTLPR genotype as well as the linear 5HTTLPR × self-blame interactions were again nonsignificant (ps > .63). The non-linear 5HTTLPR genotype × self-blame showed a trend in the expected direction, but did not reach significance [b = .06, 95% confidence interval -.03-.112, p = .11, ΔR^2 ^= .007, total R^2 ^= .09].

##### Cognitive problems and hyperactivity

The significant main effect of self-blame was again observed in predicting parent report for both Cognitive Problems [b = .30, 95% confidence interval .19-.41, p < .001, total R^2 ^= .06] and Hyperactivity [b = .12, 95% confidence interval .01-.23, p = .03, total R^2 ^= .08]. Similar to results with teacher report, all main effects of 5HTTLPR genotype (linear and non-linear coding schemes) as well as the linear 5HTTLPR genotype × self-blame interactions were not significant for both Cognitive Problems (all ps > .74) and hyperactivity (all ps > .35). The non-linear 5HTTLPR genotype × self-blame interaction was not significant for Cognitive Problems [b = .02, 95% confidence interval -.06-.09, p = .46, ΔR^2 ^= .001, total R^2 ^= .06]. It also feel shy of significance for hyperactivity [b = .06, 95% confidence interval -.01-.13, p = .10, ΔR^2 ^= .008, total R^2 ^= .08]. In sum, non-linear G × E interactions were significant in predicting teacher report of ADHD symptoms (and Hyperactivity especially), but were shy of significance when parent report was used as the dependent measure.

#### Secondary data analyses

##### Oppositionality and conduct problems

As secondary analyses, we examined the G × E interaction on the Conners' Oppositionality scale. G × E models predicting teacher report of Oppositionality yielded a main effect of self-blame [b = .15, 95% confidence interval .03-.26, p = .01, total R^2 ^= .06]. Both the linear and non-linear 5HTTLPR × self-blame interactions were non-significant, although there was a trend toward significance for the linear 5HTTLPR × self-blame interaction (p = .10). Results for parent report of Oppositionality were similar. A main effect of self-blame emerged [b = .20, 95% confidence interval .08-.31, p = .001, total R^2 ^= .07], whereas all G × E interactions involving self-blame and 5HTTLPR showed no hint of an effect (ps > .41).

For completeness, we also examined parent report of conduct disorder symptoms on the KSADS-E (we did not have an analogue of teacher report of conduct problems available for this sample). The linear main effect of 5HTTLPR approached significance [b = .08, p = .06], however all other main effects and all G × E interactions were nonsignificant (ps > .24). Overall, these results indicate that while the non-linear 5HTTLPR × self-blame interaction did not generalize to other disruptive behaviors, self-blame itself may have exert some main effects for ADHD as well as other disruptive behaviors.

##### Replication with DSM-IV symptoms

To enable comparisons with future studies that may rely on ADHD symptom scores, we here report G × E interactions using the lower powered tests with the DSM-IV ADHD Rating Scale. As with the Conners, the non-linear 5HTTLPR × self-blame interaction was significant in predicting teacher report of total ADHD symptoms [b = .13, 95% confidence interval .01-.23, p = .019, ΔR^2 ^= .03, total R^2 ^= .13] as well as for hyperactive symptoms [b = .15, 95% confidence interval .01-.28, p = .04, ΔR^2 ^= .02, total R^2 ^= .11]. The non-linear 5HTTLP × self-blame interaction predicting teacher inattentive symptoms was marginally significant, in the expected direction [b = .09, 95% confidence interval -.01-.14, p = .065, ΔR^2 ^= .02, total R^2 ^= .09]. Again, relationships between self-blame and ADHD symptoms were only positive and significant for those with the low and high 5HTTLPR activity genotypes.

For parent report of hyperactivity, we saw a trend toward non-linear 5HTTLPR × self-blame interactions [b = .07, 95% confidence interval -.02-.13, p = .15, ΔR^2 ^= .01, total R^2 ^= .10] but not for parent report of inattention (See Table4 I for complete results of effect sizes and significance for parent, teacher, and composite report for the Conners' and the ADHD Rating Scale).

**Table 4:** Appendix I

	***Effect Size ΔR***^2^	***Total R***^2^
*Teacher Report*		
Conners' ADHD Index	.02*	.13
Conners' Cognitive Problems	.008+	.08
Conners' Hyperactivity	.02*	.12
ADHD Rating Scale Total Symptoms	.03*	.13
ADHD Rating Scale Inattentive Symptoms	.02+	.09
ADHD Rating Scale Hyperactive Symptoms	.02*	.11
		
*Parent Report*		
Conners' ADHD Index	.007	.09
Conners' Cognitive Problems	.001	.06
Conners' Hyperactivity	.008	.08
ADHD Rating Scale Total Symptoms	.007	.09
ADHD Rating Scale Inattentive Symptoms	.003	.09
ADHD Rating Scale Hyperactive Symptoms	.01	.10

Effect sizes for non-linear 5HTTLPR × self-blame interactions by informant for ADHD symptoms

*Note*. * indicates significant change in R^2 ^at p < .05 uncorrected. + indicates marginally significant change in R^2 ^at p < .10 uncorrected.

#### Post-hoc data checks

##### Comorbid depression

Because of the influence of selective serotonin reuptake inhibitors and their efficacy in managing depression symptoms, we checked whether a history of comorbid depression accounted for the results. When lifetime depression symptoms (as measured via parent report on the KSADS-E) were included as a covariate, the non-linear interaction terms remained significant in predicting teacher report on the Conners' ADHD Index [b = .19, 95% confidence interval .08-.31, p = .002, ΔR^2 ^= .02, total R^2 ^= .13]. This may make sense, given that depression is often associated with only low serotonin activity and the non-linear model posited that both low and high serotonin activity genotypes would show the same relationship between self-blame and ADHD symptoms.

##### Conflict properties as a moderator

As a contrast test, we examined appraisals of conflict properties (e.g., frequency and intensity of conflict) as a moderator in the G × E analyses. While conflict properties exerted a main effect on both parent and teacher reports of ADHD symptoms (p < .01), the linear and non-linear 5HTTLPR × conflict properties interactions were not significant using either parent or teacher report (ps > .69). This suggested that results for self-blame could not be attributed to measurement artifact.

##### Age and pubertal status

The sample consisted of children and adolescents covering a wide age range (6-18 years). Serotonin systems have been suggested to function differently in pre and post-pubertal children [71]. While we covaried age in the G × E analyses, the functional serotonin literature would suggest separate analyses for pre and post-pubertal children. We therefore conducted exploratory analyses by examining G × E interactions by pubertal stage. Pubertal status was evaluated by a self-report scale used in prior work; it provides an estimated Tanner staging score that correlates about .70 with physician ratings of Tanner stage [72]. Using this measure, children were classified as belonging to one of five pubertal stages (pre-pubertal n = 16, early pubertal n = 31, mid-pubertal, n = 60, late-pubertal n = 112, and post-pubertal, n = 85). We then examined interactions separately for pre-to mid-pubertal children (n = 107) versus late to post-pubertal youth (n = 197).

These exploratory results revealed that the non-linear 5HTTLPR × self-blame interactions predicting teacher report on the Conners' ADHD Index remained significant for post and late-pubertal youth (p = .032, ΔR^2 ^= .02, total R^2 ^= .14) but only showed a trend toward significance in pre to-mid pubertal children (p = .14, ΔR^2 ^= .008, total R^2 ^= .10). While these data are preliminary, they may reflect potential differences in serotonin functioning with age and development. The influence of development on the functioning of the serotonin system, including any potential role for G × E interactions for ADHD involving serotonin system genes, remain critical questions to address in future studies.

## Discussion

When ADHD is conceptualized as emanating from the development of emotional and behavioral regulation, specific genetic and family environmental factors are likely to jointly influence ADHD outcomes in particular ways. The present report capitalized on the potential to investigate an important genetic marker for liability to emotional and behavioral dysregulation (5HTTLPR), along with a particularly salient marker of environmental risk (children's appraisals of blame in relation to inter-parental conflict).

The current study provides evidence of G × E effects for ADHD involving 5HTTLPR and youth appraisals of self-blame. Our analytic methods allowed us to examine a hypothesis previously untested at the genetic level - namely that both high and low serotonergic activity genotypes (e.g., 5HTTLPR) exert risk for ADHD symptoms. Findings from both the functional serotonin literature as well as from molecular genetic association studies have yielded seemingly conflicting findings about whether increased or reduced serotonergic activity is related to ADHD. The present results suggest that at the genetic level, *both *high and low serotonergic activity genotypes exert risk and that these risk mechanisms are modulated by salient psychosocial stressors.

The results were generally consistent across informant, although results for parent ratings were shy of the significance levels seen with teacher ratings. For teacher report, the pattern of results indicated significant non-linear 5HTTLPR × self-blame interactions for hyperactivity/impulsivity but not inattention or cognitive problems. The interactions revealed that youth appraisals of self-blame were significantly related to ADHD symptoms for children with the low activity (Lg/Lg, Lg/short, short/short) and high activity (La/La) 5HTTLPR genotypes. Those with the intermediate activity genotypes (La/Lg, La/short), on the other hand, appeared to be immune to whatever effects self blame was having on hyperactivity/impulsivity. The interaction was not accounted for by age, gender, or ethnicity, by family composition, by overall levels of conflict frequency/intensity, or by main effects of 5HTTLPR genotype or youth reports of self-blame. It was also not likely to be due to measurement artifact, because another scale (conflict properties) showed no hint of interaction.

Analysis of the symptom dimensions appeared to indicate some preliminary evidence for specificity of effects. The non-linear 5HTTLPR × self-blame interactions were non-significant or marginally significant for teacher and parent report of cognitive problems. In contrast, non-linear interactions were significant for teacher report and marginally significant for parent report of hyperactivity. The interaction again revealed that for youth with the high and low serotonin activity genotypes, the relationship between self-blame and hyperactivity was positive and significant. When examined again via the ADHD Rating Scale, a similar pattern emerged, however, the effect appeared attenuated for both parent and teacher report using DSM-IV symptom counts, perhaps due to the loss of power using the less well-distributed scores on that scale. Some potential specificity in terms of G × E effects for the ADHD symptom dimensions may make sense, as hyperactivity-impulsivity and inattention have been described as being partially separable at the genetic, neural, and temperament levels [41,73,74] as well as in recent factor analytic work [75].

The effects appeared to be somewhat specific for ADHD, as the non-linear 5HTTLPR × self-blame interactions showed no hint of an effect for oppositional defiant disorder symptoms (by teacher report) or conduct problems (by parent report). In contrast, the linear 5HTTLPR × self-blame interaction showed marginal significance for oppositional defiant disorder symptoms. If that result were to prove stronger in future work, it could indicate that only individuals with the low activity 5HTTLPR genotypes are vulnerable to development of oppositional and conduct problems [22].

Post-hoc data checks indicated that results were not generalizable to any appraisals of inter-parental conflict, as conflict properties failed to show significant moderation in the G × E analyses. Thus, there appears to be something about self-blame that is important for ADHD specifically. This is line with current work from our own laboratory (which included a portion of this sample) that indicates that among the CPIC scales, self-blame is a significant and unique predictor of ADHD symptomatology [48].

In addition, while serotonin functioning and serotonin genes have also shown association with a number of conditions, including mood disorders, the current findings were not explainable by a history of comorbid depression symptoms. Furthermore, while effects were stronger in late and post-pubertal youth, non-linear 5HTTLPR × self-blame interactions continued to show marginal significance in younger children (pre- to mid-pubertal). Thus, while developmental timing and its relationship with serotonergic functioning may be accounting for some of the effects - and will be an interesting avenue for future study - the non-linear 5HTTLPR × self-blame interactions observed here could not be fully explained developmentally.

### Implications

With regard to the genetic literature for ADHD, our results failed to replicate a main effect of 5HTTLPR genotype with ADHD symptoms that has been previously reported [76-78]. Unlike these prior studies, we genotyped the A>G substitution to create a more precise set of 5HTTLPR genotypes in regard to functionality. Using this triallelic configuration of 5HTTLPR genotype, our results are consistent with prior research, which also found no main effect of association between the triallelic formulation of 5HTTLPR and ADHD [39]. Furthermore, while our results did not support a main effect of 5HTTLPR, these results add to recent evidence that serotonin genetic risk as indexed by 5HTTLPR genotype and disruptions in the family environment interact together to predict deficits in behavioral and emotional regulation [79,80]. Overall, these results complement growing evidence suggesting that 5HTTLPR confers liability for ADHD that is activated in particular environments, rather than conferring risk for ADHD directly.

The overall results are also suggestive of a potential heterozygote advantage, which has been suggested as a potential explanation for some diseases, including mental disorders [81]. However, a recent G × E study examining 5HTTLPR and psychosocial risk indicated effects in the opposite direction: only those with heterozygote 5HTTLPR genotypes showed increased violent behavior in adulthood within the context of an adverse rearing environment [82]. It may be the case that any 'heterozygote advantage' in 5HTTLPR may be modulated by developmental stage. In the present study, concurrent negative outcomes (i.e., increased ADHD behaviors) were associated with interactions between low and high 5HTTLPR activity and self-blame, whereas the prior G × E study [82] evidenced heterozygote "disadvantage" for outcomes later in life. In line with this, the serotonin system has been shown to function differently in children and adults in regard to its association with impulsive and aggressive behavior. Further, our main outcome variables involved ADHD and oppositionality symptoms measured in childhood and adolescence, compared with aggressive and violent behavior measured in adulthood, which was examined as outcome measures in this prior G × E research [82]. It is possible that the heterozygote genotype may differentially exert risk or protect from negative outcomes depending upon developmental stage (childhood and adolescence versus adulthood) as well as the type of behavior being assessed (inattention, hyperactivity-impulsivity versus aggression and violence).

### Limitations

Certain limitations are important to note. First, we did not have parent DNA available for the majority of our sample, thus the use of family-based analyses was not possible. While unlikely, we cannot rule out population stratification effects. Second, because our sample is cross-sectional, we could not examine the longitudinal relationships between appraisals of self-blame and ADHD symptoms. It may be the case that more frequent inter-parental conflict is the result of having a child with more severe ADHD symptoms and that over time, children view themselves (perhaps correctly) as being responsible for their parents' marital problems. As is always the case with single studies of genetic or environmental effects, our findings may be false-positives. Thus, replication of these results in other samples is necessary.

The influence of development on the functioning of the serotonin system, including any potential role for G × E interactions for ADHD involving serotonin system genes, remains in need of further investigation in more costly studies, which may be justified by results such as the current one. In addition, the influence of age regarding comprehension of the 48-item CPIC remains in need of further exploration. While we took several steps to assure correct comprehension and completion of the CPIC in younger children, future work extending on these results may be well-served by considering the CPIC-Y designed for younger children [61].

Overall, our study is among the first to examine relationships between 5HTTLPR and ADHD as well as interaction effects using the triallelic genotype configuration. Results suggest that both the low- and high-activity 5HTTLPR genotypes increase risk for ADHD symptoms within the context of higher levels of youth self-blame in relation to their parents' marital conflict.

## Competing interests

The authors declare that they have no competing interests.

## Authors' contributions

MN formulated study hypothesis, participated in data collection, carried out the analyses, and wrote the manuscript draft. KF oversaw genotyping procedures, obtained funding, aided in hypothesis generation, and provided manuscript revision. IW provided statistical consultation, formulated data analytic plan, and provided manuscript revision. KJ completed genotyping assays on the sample. JTN advised on hypothesis formulation, obtained funding, assisted with data analysis and manuscript revision. All authors read and approved the final manuscript.

## References

[B1] Nigg JT Hinshaw SP Huang-Pollock C Cicchetti D, Cohen DJ Disorders of attention and impulse regulation Developmental Psychopathology, Risk, disorder, and adaptation 2006 3 2 Hoboken, NJ: John Wiley & Sons 358 403

[B2] Rutter M Silberg J Gene-environment interplay in relation to emotional and behavioral disturbance Annu Rev Psychol 2002 53 463 490 10.1146/annurev.psych.53.100901.135223 11752493

[B3] Bergen SE Gardner CO Kendler KS Age-related changes in heritability of behavioral phenotypes over adolescence and young adulthood: A meta-analysis Twin Res Human Genet 2007 10 423 433 10.1375/twin.10.3.423 17564500

[B4] Faraone SV Perlis RH Doyle AE Smoller JW Goralnick JJ Holmgren MA Sklar P Molecular genetics of ADHD Biol Psychiatry 2005 57 1313 1323 10.1016/j.biopsych.2004.11.024 15950004

[B5] Gizer IR Ficks C Waldman ID Candidate gene studies of ADHD: A meta-analytic review Hum Genet 2009 126 51 90 10.1007/s00439-009-0694-x 19506906

[B6] Purcell S Variance component models for gene-environment interaction in twin analysis Twin Res 2002 5 554 571 10.1375/136905202762342026 12573187

[B7] Banerjee TD Middleton F Faraone SV Environmental risk factors for attention-deficit hyperactivity disorder Acta Pediatri 2007 96 1269 74 10.1111/j.1651-2227.2007.00430.x 17718779

[B8] Nigg JT What causes ADHD? Understanding what goes wrong and why 2006 New York: The Guilford Press

[B9] Pennington BF McGrath LM Rosenberg J Barnard H Smith SD Willcutt EG Friend A DeFries JC Olson RK Gene X environment interactions for reading disability and attention-deficit hyperactivity disorder Dev Psychol 2009 45 77 89 10.1037/a0014549 19209992 PMC2743891

[B10] Spencer T Biederman J Wilens T Pharmacoptherapy of attention deficit hyperactivity disorder Child Adolesc Psychiatr Clin N Am 2000 9 77 97 10674191

[B11] Nigg JT Casey BJ An integrative theory of attention-deficit hyperactivity disorder based on the cognitive and affective neurosciences Dev Psychopathol 2006 17 785 806 10.1017/S0954579405050376 16262992

[B12] Evans J Platts S Lightman S Nutt D Impulsiveness and the prolactin response to d-flenfluramine Psychopharmacology 2000 149 147 152 10.1007/s002139900361 10805609

[B13] Moffitt TE Brammer GL Caspi A Fawcett JP Raleigh M Yuwiler A Silva P Whole blood serotonin relates to violence in an epidemiological study Biol Psychiatry 1998 43 446 457 10.1016/S0006-3223(97)00340-5 9532350

[B14] Depue RA Spoont MR Conceptualizing a serotonin trait: A behavioral dimension of constraint Ann NY Acad Sci 1980 487 47 62 10.1111/j.1749-6632.1986.tb27885.x 2436540

[B15] Frankle WG Huang Y Hwang DR Talbot PS Slifstein M Van Heertum R Abi-Dargham A Laruelle M Comparative evaluation of serotonin transporter radioligands 11C-DASB and 11C-McN 5652 in healthy humans J Nucl Med 2004 45 682 694 15073266

[B16] Lesch KP Bengel D Heils A Sabol SZ Greenberg BD Petri S Benjamin J Muller CR Hamer DH Murphy DL Association of anxiety-related traits with a polymorphism in the serotonin transporter gene regulatory region Science 1996 274 1527 1531 10.1126/science.274.5292.1527 8929413

[B17] Greenberg BD Tolliver TJ Huang SJ Li Q Bengel D Murphy DL Genetic variation in the serotonin transporter promoter region affects serotonin uptake in human blood platelets Am J Med Genet 1999 88 83 87 10.1002/(SICI)1096-8628(19990205)88:1<83::AID-AJMG15>3.0.CO;2-0 10050973

[B18] Bennet A Lesch KP Heils A Long JC Lorenz JG Schoaf SE Champoux M Suomi SJ Linnoila MV Higley JD Early experience and serotonin transporter gene interact to influence primate CNS function Mol Psychiatry 2002 7 118 122 10.1038/sj/mp/4000949 11803458

[B19] Manuck SB Flory JD Ferrell RE Muldoon RF Socio-economic status covaries with central nervous system serotonergic responsivity as a function of allelic variation in the serotonin transporter gene-linked polymorphic region Psychoneuroendocrinology 2004 29 651 668 10.1016/S0306-4530(03)00094-5 15041087

[B20] Sonuga-Barke EJ Lasky-Su J Neale B Oades R Chen W Franke B Buitelaar J Banaschewski T Ebstein R Gill M Anney R Miranda A Mulas F Roeyers H Rothenberger A Sergeant J Steinhausen HC Thompson M Asherson P Faraone SV Does parental expressed emotion moderate genetic effects in ADHD? An exploration using a genome wide association scan Am J Med Genet B Neuropsychiatr Genet 2008 147B 1359 68 10.1002/ajmg.b.30860 18846501

[B21] Hallikainen T Saito T Lachman HM Volavka J Pohjalainen T Ryynanen OP Kauhanen J Syvalahti E Hietala J Tiihonen J Association between low activity serotonin transporter promoter genotype and early-onset alcoholism with habitual violent impulsive behavior Mol Psychiatry 1999 4 385 388 10.1038/sj.mp.4000526 10483057

[B22] Beitchman JH Baldassarra L Mik H DeLuca V King N Bender D Ehtesham S Kennedy JL Serotonin transporter polymorphisms and persistent, pervasive childhood aggression Am J Psychiatry 2006 163 1103 1105 10.1176/appi.ajp.163.6.1103 16741214

[B23] Haberstick BC Smolen A Hewitt JK Family-based association test of 5HTTLPR and aggressive behavior in a general population sample of children Biol Psychiatry 2006 59 836 843 10.1016/j.biopsych.2005.10.008 16412987

[B24] Sakai JT Young SE Stallings MC Timberlake D Smolen A Stelter GL Crowley TJ Case-control and within-family tests for an association between conduct disorder and 5HTTLPR Am J Med Genet B Neuropsychiatr Genet 2006 141B 825 832 10.1002/ajmg.b.30278 16972235

[B25] Kruesi MJ Rapoport JL Hamburger S Hibbs E Potter WZ Lenane M Brown GL Cerebrospinal fluid monamine metabolites, aggression, and impulsivity in disruptive behavior disorders of children and adolescents Arch Gen Psychiatry 1990 47 419 426 1691910 10.1001/archpsyc.1990.01810170019003

[B26] Kruesi MJ Hibbs ED Zahn TP Keysor CS Hamburger SD Bartko JJ Rapoport JL A 2-year prospective follow-up study of children and adolescents with disruptive behavior disorders: Prediction by cerebrospinal fluid 5-hydroxyinoleacetic acid, homovanillic acid, and autonomic measures? Arch Gen Psychiatry 1992 49 429 435 1376104 10.1001/archpsyc.1992.01820060009001

[B27] Clarke RA Murphy DL Constantino JN Serotonin and externalizing behavior in young children Psychiatry Res 1999 86 29 40 10.1016/S0165-1781(99)00022-0 10359480

[B28] Flory JD Newcorn JH Miller C Harty S Halperin JM Serotonergic function in children with attention-deficit hyperactivity disorder: Relationship to later antisocial personality disorder Br J Psychiatry 2007 190 410 414 10.1192/bjp.bp.106.027847 17470955

[B29] Stoff DM Pasatiempo AP Yeung J Cooper TB Bridger WH Rabinovich H Neuroendocrine responses to challenge with d-fenfluramine and aggression in disruptive behavior disorders of children and adolescents Psychiatry Res 1992 43 263 276 10.1016/0165-1781(92)90059-C 1438624

[B30] Castellanos FX Elia J Kruesi MJ Gulotta CS Mefford IN Potter WZ Ritchie GF Rapoport JL Cerebrospinal fluid monoamine metabolites in boys with a attention-deficit hyperactivity disorder Psychiatry Res 1994 52 305 316 10.1016/0165-1781(94)90076-0 7527565

[B31] Halperin JM Sharma V Siever LJ Schwartz ST Matier K Wornell G Newcorn JH Serotonergic function in aggressive and nonaggressive boys with attention-deficit hyperactivity disorder Am J Psychiatry 1994 151 243 248 8296897 10.1176/ajp.151.2.243

[B32] Coccaro EF Siever LJ Klar HM Maurer G Cochrane K Cooper TB Mohs RC Davis KL Serotonergic studies in patients with affective and personality disorders: Correlates with suicidal and impulsive aggressive behavior Arch Gen Psychiatry 1989 46 587 599 2735812 10.1001/archpsyc.1989.01810070013002

[B33] O'Keane V Maloney E O'Neil H O'Connor A Smith C Dinan TG Blunted prolactin response to d-fenfluramine in sociopathy: Evidence for subsensitivity of central serotonergic function Br J Psychiatry 1992 160 643 646 10.1192/bjp.160.5.643 1591573

[B34] Manuck SB Flory JD Muldoon MF Ferrell RE Central nervous system serotonergic responsivity and aggressive disposition in men Physiol Behav 2002 77 705 709 10.1016/S0031-9384(02)00922-8 12527023

[B35] Retz W Retz-Jungunger P Supprian T Thome J Rosler M Association with serotonin transporter gene polymorphism with violence: Relation with personality disorders, impulsivity, and childhood ADHD psychopathology Behav Sci Law 2004 22 415 425 10.1002/bsl.589 15211560

[B36] Nakamura M Ueno S Sano A Tanabe H The human serotonin transporter gene linked polymorphism (5HTTLPR) shows ten novel allelic variants Mol Psychiatry 2000 5 32 38 10.1038/sj.mp.4000698 10673766

[B37] Hu XZ Lipsky RH Zhu G Akhtar LA Taubman J Greenberg BD Xu K Arnold PD Richter MA Kennedy JL Murphy DL Goldman D Serotonin transporter promoter gain-of-function genotypes are linked obsessive-compulsive disorder Am J Hum Genet 2006 78 815 826 10.1086/503850 16642437 PMC1474042

[B38] Kraft JM Slager SL McGrath PJ Hamilton SP Sequence analysis of the serotonin transporter and associations with antidepressant response Biol Psychiatry 2005 58 374 381 10.1016/j.biopsych.2005.04.048 15993855

[B39] Wigg KG Takhar A Ickowicz A Tannock R Kennedy JL Pathare T Malone M Schachar R Barr C Gene for the serotonin transporter and ADHD: No association with two functional polymorphisms Am J Med Genet B Neuropsychiatr Genet 2006 141B 566 570 10.1002/ajmg.b.30247 16856124

[B40] Oades RD Lasky-Su J Christiansen H Faraone SV Sonuga-Barke EJS Banaschewski T Chen W Anney RJL Buitelaar JK Ebstein RP Franke B Gill M Miranda A Roeyers H Rothenberger A Sergeant JA Steinhausen H Taylor EA Thompson M Asherson P The influence of serotonin-and other genes on impulsive behavioral aggression and cognitive impulsivity in children with attention-deficit hyperactivity disorder (ADHD): Findings from a family-based association test (FBAT) analysis Behav Brain Funct 2008 4 48 10.1186/1744-9081-4-48 18937842 PMC2577091

[B41] Nikolas M Burt SA Genetic and environmental influences on ADHD symptom dimensions of inattention and hyperactivity: A meta-analysis J Abnorm Psychology 2010 119 1 17 10.1037/a0018010 20141238

[B42] Retz W Freitag CM Retz-Junginger P Wenzler D Schneider M Kissling C Thome J Rosler M A functional serotonin transporter gene polymorphism increases ADHD symptoms in delinquents: Interaction with adverse child environment Psychiatry Res 2008 158 123 131 10.1016/j.psychres.2007.05.004 18155777

[B43] Arnsten AF Goldman-Rakic PS Noise impairs prefrontal cortical cognitive function in monkeys: Evidence for a hyperdopaminergic mechanism Arch Gen Psychiatry 1998 55 362 368 10.1001/archpsyc.55.4.362 9554432

[B44] Grych JH Fincham FD Marital conflict and children's adjustment: A cognitive-contextual framework Psychol Bull 1990 108 267 290 10.1037/0033-2909.108.2.267 2236384

[B45] Cummings EM Davies PT Children and marital conflict: The impact of marital dispute and resolution 1994 New York: The Guilford Press

[B46] Grych JH Fincham FD Jouriles EN McDonald RN Interparental conflict and child adjustment: Testing the mediational role of appraisals in the cognitive-contextual framework Child Dev 2000 71 1648 1661 10.1111/1467-8624.00255 11194263

[B47] Jouriles EN Collazos Spiller L Stephens N McDonald R Swank P Variability in adjustment of child of battered women: The Role of child appraisals of interparental conflict Cognit Ther Res 2000 24 233 249 10.1023/A:1005402310180

[B48] Counts CA Nigg JT Stawicki JA Rappley MD Von Eye A Family adversity in DSM-IV ADHD Combined and Inattentive subtypes as associated disruptive behavior problems J Am Acad Child Adolesc Psychiatry 2005 44 690 698 10.1097/01.chi.0000162582.87710.66 15968238

[B49] Gerard JM Beuhler C Franck K Anderson O In the eyes of the beholder: Cognitive appraisals as mediators of the association between interparental conflict and youth maladjustment J Fam Psychol 2005 19 376 384 10.1037/0893-3200.19.3.376 16221018

[B50] Skopp NA McDonald R Manke B Jouriles EN Siblings in domestically violent families: Experiences of interparent conflict and adjustment problems J Fam Psychol 2005 19 324 333 10.1037/0893-3200.19.2.324 15982110

[B51] Buehler C Lange G Franck KL Adolescents' cognitive and emotional responses to marital hostility Child Dev 2007 78 775 789 10.1111/j.1467-8624.2007.01032.x 17517004

[B52] Wymbs BT Pelham WE Molina BSG Gnagy EM Mother and adolescent reports of interparental discord among parents of adolescents with and without attention-deficit/hyperactivity disorder J Emot Behav Disord 2008 16 29 41 10.1177/1063426607310849 20016758 PMC2794134

[B53] Grych JH Seid M Fincham FD Assessing marital conflict from the child's perspective: The Children's Perception of Interparental Conflict Scale Child Dev 1992 63 558 572 10.2307/1131346 1600822

[B54] Cummings EM Davies PT Simpson KS Marital conflict, gender, and children's appraisals and coping efficacy as mediators of child adjustment J Fam Psychol 1994 8 141 149 10.1037/0893-3200.8.2.141

[B55] Davies PT Sturge-Apple ML Cicchetti D Cummings EM Adrenocorticol underpinnings of children's psychological reactivity to interparental conflict Child Dev 2008 79 1693 1706 10.1111/j.1467-8624.2008.01219.x 19037943 PMC2597091

[B56] El-Sheikh M Kouros CD Erath S Cummings EM Keller P Staton L Interactions among marital conflict, sympathetic, and parasympathetic nervous system activity in the prediction of children's externalizing problems Monogr Soc Res Child Dev 2009 74 19 34 10.1111/j.1540-5834.2009.00503.x PMC2918238 19302676

[B57] Rhoades KA Children's responses to interparental conflict: A meta-analysis of their associations with child adjustment Child Dev 2008 79 1942 1956 10.1111/j.1467-8624.2008.01235.x 19037959 PMC2597571

[B58] Connors CK Connors' Rating Scales - Revised 1997 Toronto, Ontario, Canada: Multi-Health Systems, Inc

[B59] DuPaul GJ Power TJ Anastopoulos AD Reid R The ADHD rating scale -IV:Checklists, norms, and clinical interpretation 1998 New York: Guilford Press

[B60] Puig-Antich J Ryan N The Schedule for Affective Disorders and Schizophrenia for School-Age Children (Kiddie-SADS) 1986 Pittsburgh, Pennsylvania. Western Psychiatric Institute and Clinic

[B61] McDonald R Grych JH Young children's appraisals of interparental confict and links with adjustment problems J Fam Psychol 2006 20 88 99 10.1037/0893-3200.20.1.88 16569093

[B62] Wechsler D Handbook for the Wechsler Individual Achievement Test 2005 2 London: Harcourt Asessment

[B63] Nigg JT Nikolas M Miller T Burt SA Klump KL von Eye A Factor structure of the Child Perception of Interparental Conflict Scale for studies of youth with externalizing behavior problems Psychol Assess 2009 19719356 10.1037/a0016564 PMC2818811

[B64] Porter B O'Leary KD Marital discord and child behavior problems J Abnorm Child Psychol 1980 8 287 295 10.1007/BF00916376 7410730

[B65] Meulenbelt I Droog S Trommelen GJ Boomsma DI Slagboom P High-yield non-invasive human genomic DNA isolation method for genetic studies in geographically dispersed families and populations Am J Hum Genet 1995 57 1252 1254 7485180 PMC1801361

[B66] Eaves LJ Genotype × environment interaction in psychopathology: Fact or artifact? Twin Res Hum Genet 2006 9 1 8 10.1375/183242706776403073 16611461

[B67] Duncan GJ Dowsett CJ Claessens A Magnuson J Huston AC Klebanoc P Pagani L Feinstein L Engel M Brooks-Gunn J Sexton H Duckworth K Japel C School readiness and later achievement Dev Psychol 2007 43 1428 1446 10.1037/0012-1649.43.6.1428 18020822

[B68] Breslau J Lane M Sampson N Kessler RC Mental disorders and subsequent educational attainment in a US National Sample Journal of Psychiatr Res 2008 42 708 716 10.1016/j.jpsychires.2008.01.016 PMC2748981 18331741

[B69] Breslau N Breslau J Peterson E Miller E Lucia VC Bonhert K Nigg J Shifts in academic achievement from age 11 to age 17: The role of early development of attention problems Pediatrics 2009 123 1472 1476 10.1542/peds.2008-1406 19482756 PMC2778327

[B70] Keppel G Wickens TD Design and analysis: A researcher's handbook 2004 4 Upper Saddle River, NJ: Pearson/Prentice Hall

[B71] Spear LP The adolescent brain and age-related behavioral manifestations Neurosci Biobehav Rev 2000 24 417 463 10.1016/S0149-7634(00)00014-2 10817843

[B72] Angold A Costello EJ Erkanli A Worthman CP Pubertal changes in hormone levels and depression in girls Psych Medicine 1999 29 1043 1053 10.1017/S0033291799008946 10576297

[B73] Sonuga-Barke EJS Causal models of attention-deficit/hyperactivity disorder: From common simple deficits to multiple developmental pathways Biol Psychiatry 2005 57 1231 1238 10.1016/j.biopsych.2004.09.008 15949993

[B74] Martel MM Nigg JT Child ADHD and personality/temperament traits of reactive and effortful control, resiliency, and emotionality J Child Psychol Psychiatry 2006 47 1175 1183 10.1111/j.1469-7610.2006.01629.x 17076757

[B75] Lahey BB Rathouz PJ Van Hulle C Urbano RC Krueger RF Applegate B Garriock HA Chapman DA Waldman ID Testing structural models of DSM-IV symptoms of common forms of child and adolescent psychopathology J Abnorm Child Psychol 2008 36 187 206 10.1007/s10802-007-9169-5 17912624

[B76] Manor I Eisenberg J Tyano S Sever Y Cohen H Ebstein RP Kotler M Family-based association study of the serotonin transporter promoter polymorphism (5-HTTLPR) in attention-deficit hyperactivity disorder Am J Med Genet 2001 105 91 95 10.1002/1096-8628(20010108)105:1<91::AID-AJMG1069>3.0.CO;2-V 11425009

[B77] Seeger G Schloss P Schmidt MH Functional polymorphism within the promoter of the serotonin transporter gene is associated with severe hyperkinetic disorders Mol Psychiatry 2001 6 235 238 10.1038/sj.mp.4000820 11317229

[B78] Kent L Doerry U Hardy E Parmar R Gingell K Hawi Z Kirley A Lowe N Fitzgerald M Gill M Craddock N Evidence that variation at the serotonin transporter gene influences susceptibility to attention deficit hyperactivity disorder (ADHD): Analysis and pooled analysis Mol Psychiatry 2002 7 908 912 10.1038/sj.mp.4001100 12232786

[B79] Barry RA Kochanska G Philibert RA GxE interaction in the organization of attachment: Mothers' responsiveness as a moderator of children's genotypes J Child Psychol Psychiatry 2008 49 1313 1320 10.1111/j.1469-7610.2008.01935.x 19120710 PMC2688730

[B80] Pauli-Pott U Friedl S Hinney A Hebebrand J Serotonin transporter gene polymorphism (5HTTLPR), environmental conditions, and developing negative emotionality and fear in early childhood J Neural Transm 2009 116 503 512 10.1007/s00702-008-0171-z 19137235

[B81] Keller MC Miller G Resolving the paradox of common, harmful, heritable mental disorders: Which evolutionary genetic models work best? Behav Brain Sci 2006 29 385 404 17094843 10.1017/S0140525X06009095

[B82] Reif A Rosler M Schneider M Eujen A Kissling C Jacob CP Retz-Junginger P Thome J Lesch KP Retz W Nature and nuture predispose to violent behavior: Serotonergic genes and adverse child environment Neuropsychopharmacology 2007 32 2375 2383 10.1038/sj.npp.1301359 17342170

